# Multiplatform molecular test performance in indeterminate thyroid nodules

**DOI:** 10.1002/dc.24564

**Published:** 2020-08-07

**Authors:** Mark A. Lupo, Ann E. Walts, J. Woody Sistrunk, Thomas J. Giordano, Peter M. Sadow, Nicole Massoll, Ryan Campbell, Sara A. Jackson, Nicole Toney, Christina M. Narick, Gyanendra Kumar, Alidad Mireskandari, Sydney D. Finkelstein, Shikha Bose

**Affiliations:** ^1^ Thyroid & Endocrine Center of Florida Sarasota Florida USA; ^2^ Cedars‐Sinai Medical Center Los Angeles California USA; ^3^ Jackson Thyroid & Endocrine Clinic Jackson Mississippi USA; ^4^ University of Michigan Ann Arbor Michigan USA; ^5^ Massachusetts General Hospital and Harvard Medical School Boston Massachusetts USA; ^6^ University of Arkansas for Medical Sciences Little Rock Arkansas USA; ^7^ Interpace Diagnostics Parsippany New Jersey USA

**Keywords:** indeterminate thyroid nodules, malignancy, molecular test, outcomes

## Abstract

**Background:**

Approximately 25% of thyroid nodule fine‐needle aspirates (FNAs) have cytology that is indeterminate for malignant disease. Accurate risk stratification of these FNAs with ancillary testing would reduce unnecessary thyroid surgery.

**Methods:**

We evaluated the performance of an ancillary multiplatform test (MPTX) that has three diagnostic categories (negative, moderate, and positive). MPTX includes the combination of a mutation panel (ThyGeNEXT®) and a microRNA risk classifier (ThyraMIR®). A blinded, multicenter study was performed using consensus histopathology diagnosis among three pathologists to validate test performance.

**Results:**

Unanimous consensus diagnosis was reached in 197 subjects with indeterminate thyroid nodules; 36% had disease. MPTX had 95% sensitivity (95% CI,86%‐99%) and 90% specificity (95% CI,84%‐95%) for disease in prevalence adjusted nodules with Bethesda III and IV cytology. Negative MPTX results ruledout disease with 97% negative predictive value (NPV; 95% CI,91%‐99%) at a 30% disease prevalence, while positive MPTX results ruledin high risk disease with 75% positive predictive value (PPV; 95% CI,60%‐86%). Such results are expected in four out of five Bethesda III and IV nodules tested, including *RAS* positive nodules in which the microRNA classifier was useful in rulingin disease. 90% of mutation panel false positives were due to analytically verified *RAS* mutations detected in benign adenomas. Moderate MPTX results had a moderate rate of disease (39%, 95% CI,23%‐54%), primarily due to *RAS* mutations, wherein the possibility of disease could not be excluded.

**Conclusions:**

Our results emphasize that decisions for surgery should not solely be based on *RAS* or *RAS*‐like mutations. MPTX informs management decisions while accounting for these challenges.

## 1 INTRODUCTION

Although thyroid nodules are extremely common, thyroid cancer is relatively infrequent, with only approximately 16 new cases diagnosed per 100 000 adults per year in the United States.^1^ Given that most thyroid nodules are benign, it is beneficial to preoperatively distinguish nodules that are likely benign from those that are likely malignant in order to minimize unnecessary surgery for benign nodules and reserve surgery for clinically significant malignancy. This risk assessment is also important for preoperative patient counseling, discussion, and surgical planning.

Thyroid nodules typically undergo ultrasound‐guided fine‐needle aspiration (FNA) biopsy for cytopathologic evaluation and malignancy assessment. Using The Bethesda System for Reporting Thyroid Cytopathology (TBSRTC), cytology findings are classified into one of six diagnostic categories, each with an assigned risk of malignancy.^2^ The Bethesda categories are (I) non‐diagnostic or unsatisfactory, (II) benign, (III) atypia of undetermined significance (AUS) or follicular lesion of undetermined significance (FLUS), (IV) follicular neoplasm (FN) or suspicious for a follicular neoplasm (SFN), (V) suspicious for malignancy, and (VI) malignant. Approximately 25% of thyroid nodule aspirates are classified as Bethesda III, IV, or V, which are considered indeterminate for malignancy.^3^ The average rate of malignancy (ROM) associated with the Bethesda III, IV, and V categories is 18% (range 6%‐30%), 25% (range 10%‐40%), and 60% (range 45%‐75%), respectively.^2^ As a result, many patients with benign nodules undergo surgery that could potentially be avoided.

Molecular tests have been increasingly used in the clinical setting as adjuncts to further risk stratify nodules with indeterminate cytology. The objective is to distinguish patients who are more likely to benefit from conservative management (ie, continued surveillance) from those who are more likely to benefit from surgical intervention. Some commercially available molecular tests, such as ThyroSeq® and ThyGeNEXT®, are oncogenic driver mutation panels.^4^, ^5^ Strong driver mutations that are highly predictive of malignancy, such as *BRAF V600E* mutations, *RET* fusions, and *TERT* promoter mutations, have proven useful in surgical decision making.^6^, ^7^, ^8^, ^9^, ^10^, ^11^, ^12^, ^13^ However, the most common mutations in indeterminate nodules are *RAS*, which are weak driver mutations with a lower PPV. In recent studies of *RAS* mutations, the PPV ranged from only 10% to 37% across multiple institutions.^14^, ^15^ Although some have reported a higher PPV for *RAS* mutations, others have suggested that this higher PPV may reflect *RAS* performance in distinct benign and malignant histopathologic subtypes included in those studies.^16^ In addition, many other mutations and fusions that are included in commercial mutation panels occur at very low frequencies, making their predictive value for malignancy difficult to study and consequently uncertain.

RNA‐based risk classifier approaches have also been increasingly used in the clinical setting to risk stratify thyroid nodules with indeterminate cytology. A messenger RNA‐based genomic sequencing classifier (GSC, Afirma®) can ruleout the need for surgery through its reported high NPV (96%) but cannot effectively rulein the need for surgery due to its suboptimal PPV (47%).^17^ In contrast, a multiplatform test (MPT) approach that combines a mutation panel test (ThyGenX®) and a microRNA risk classifier test (ThyraMIR®) has been shown to provide both high NPV and high PPV for malignancy and have clinical utility.^18^, ^19^ Negative MPT test results have been associated with the same low rate (11%) of nodule surgical resection as other tests that effectively ruleout the need for surgery, including benign cytology and GSC.^19^, ^20^ Positive MPT results have been associated with high rates (84%) of surgical resection, consistent with a test that effectively rulesin the need for surgery.^19^

In the current version of MPT, designated MPTX, an analytically validated expanded, next generation sequencing test (ThyGeNEXT) is used in combination with the microRNA risk classifier test (ThyraMIR).^4^, ^21^, ^22^ The expanded mutation panel includes *NTRK* and *ALK* fusions that have targeted therapies, as well as *TERT* and *RET* proto‐oncogene mutations that are markers of aggressive disease.^6^, ^23^, ^24^, ^25^, ^26^, ^27^, ^28^ In MPTX testing, samples with no detectable mutational change and those that have weak driver mutations are further risk stratified using the microRNA classifier, which incorporates two thresholds for malignancy risk.^29^ The first threshold was designed to optimize sensitivity for malignancy while the second threshold was designed to maximize specificity.^18^, ^29^ MPTX results are reported as one of three categories (negative, moderate, or positive) based on results of the mutation panel and microRNA risk classifier thresholds. Sistrunk et al 2020 reported the cumulative, cancer‐free survival associated with these categories in patients who underwent MPTX in a clinical setting.^30^ However, the performance characteristics of these three categories have not been previously reported in a validation study using a histopathology gold standard to evaluate test performance.

We evaluated the performance of MPTX in a blinded validation study of thyroid nodules with indeterminate cytology that later underwent surgical resection. Unanimous consensus histopathology diagnosis among three pathologists was used as the gold standard for test performance to control for inter‐observer variability among pathologists.^2^, ^31^, ^32^ MPTX performance was also evaluated after the proportions of distinct histopathologic subtypes observed in our study were prevalenceadjusted to match those reported in a different prospective study that examined similar histopathologic subtypes and mutations.^5^

## 2 MATERIALS AND METHODS

### 2.1 FNA Samples from subjects

In our multi‐center retrospective study, MPTX was performed on FNAs archived as cytology slides from nonconsecutive subjects with indeterminate cytology who did not have past MPTX testing. Four independent medical centers (Thyroid & Endocrine Center of Florida, Sarasota, FL; Cedars‐Sinai Medical Center, Los Angeles, CA; Jackson Thyroid & Endocrine Clinic, Jackson, MS; University of Arkansas for Medical Sciences, Little Rock, AR) were invited to contribute a maximum of two representative Papanicolau or Diff–Quik stained cytology slides from each thyroid FNA performed clinically and reported as having indeterminate cytology (Bethesda III, IV, V) at their institution. A corresponding representative follow‐up formalin fixed, paraffin embedded, H&E stained tissue section from the surgically resected nodule was also provided. Only slides with adequate thyroidal epithelial cellularity (ie, at least 80 cells per slide) that had been archived for fewer than 10 years and were from patients greater than 18 years of age were accepted. The study was approved by a central independent ethics review board with informed consent waived due to minimal risk (Advarra IRB #33697).

### 2.2 Molecular testing

Prior to the present study, Interpace Diagnostics had performed molecular testing on a cohort of archived cytology slides from thyroid nodule FNAs to assess DNA and RNA degradation over time after cytology fixation. Based on these results, it was expected that approximately 23% of cytology slides that had been archived for a median of 5 years would fail to provide molecular results due to DNA and RNA degradation(SD Finkelstein, unpublished observation).

In the current study, all molecular testing was performed by Interpace Diagnostics using archived thyroid cytology slides that had been collected by FNAs performed and processed in accordance with usual clinical and laboratory practice. Interpace was blind to the histopathology outcomes of subjects when molecular testing was performed. All molecular testing was performed using standard clinical procedures for the ThyGeNEXT mutation panel and ThyraMIR microRNA risk classifier commercial tests (Interpace Diagnostics). All molecular test results were stored and finalized in a secure laboratory information system (LIMS) database that was password protected and separated from that which harbored de‐identified baseline characteristics and follow‐up histopathology diagnoses of subjects.

The expanded mutation panel test (ThyGeNEXT) utilizes targeted next‐generation sequencing (NGS) (MiSeq, Illumina) to detect messenger RNA fusion transcripts and DNA mutation variants listed in Table 1. For a positive variant call, a specimen was required to contain at least 3% *BRAF V600E*, 10% *GNAS*, or 5% other individual DNA variants in the panel. Although the ThyGeNEXT assay has been previously analytically validated,^4^ the accuracy of the NGS assay for detecting individual *RAS* mutations was additionally verified using competitive allele‐specific PCR testing (TaqMan Mutation Detection Assay, Thermo Fisher Scientific) of randomly selected samples in the study that had (n = 35) or did not have (n = 35) *RAS* mutations. The microRNA risk classifier test (ThyraMIR) was performed using a validated panel of 10 specific microRNAs tested using quantitative RT‐PCR (QuantStudio) to evaluate microRNA expression levels in relation to one another.^18^, ^33^ The panel of microRNAs tested is listed in Table 1.

**TABLE 1:** The multiplatform test (MPTX) showing mutations and messenger RNA fusion transcripts (ThyGeNEXT) and microRNAs (ThyraMIR)

Expanded mutation panel (ThyGeNEXT)		microRNA risk classifier(ThyraMIR)
DNA variant	Fusions (n) and mRNA	microRNA
*BRAF* ^a^	*BRAF* (3)^b^	miR‐31‐5p
*ALK*	*ALK* (2)	miR‐29b‐1‐5p
*GNAS*	*NTRK* (8)	miR‐138‐1‐3p
*HRAS*	*PPARg* (5)	miR‐139‐5p
*KRAS*	*RET* (14)^b^	miR‐146b‐5p
*NRAS*	*THADA* (5)	miR‐155
*PIK3CA*	*NKX2.1*	miR‐204‐5p
*PTEN*	*PAX8*	miR‐222‐3p
*RET* ^b^	*TBP*	miR‐375
*TERT* promoter^b^	*USP33*	miR‐551b‐3p

Abbreviation: mRNA, messenger RNA.

^a^ *BRAF*V600E is a strong driver mutation, while *BRAF*K601E is a weak driver mutation.

^b^ Strong driver mutation.

### 2.3 MPTX test results

MPTX test results were recorded as negative, moderate, or positive in accordance with Interpace's standard clinical procedures, blind to histopathology outcomes. All MPTX test results were stored in the LIMS database that was password protected and separated from that which harbored de‐identified histopathology outcomes. In MPTX, samples with strong driver mutations do not undergo microRNA risk classification, while those with weak drivers or no detectable mutations are further risk stratified by microRNA levels. MPTX is resulted as negative when no mutations are detected and the microRNA test is negative (Level‐1); as positive when a strong driver mutation is detected or when the microRNA test is positive (Level‐3); and as moderate when a weak driver mutation is detected and the microRNA test is negative or moderate (Levels 1‐2), or when no mutations are detected and the microRNA test is moderate (Level‐2). The development of negative, moderate, and positive microRNA levels (Levels 1‐3) have been previously described, with the current study serving as a validation of this approach.^18^, ^29^ The threshold for negative MPTX results was designed to optimize sensitivity, while the threshold for positive MPTX results was designed to maximize specificity. In MPTX, *BRAFV600E*, *TERT*, and *RET* mutations and *BRAF* and *RET* related fusions are categorized as strong oncogenic drivers based on their established high PPV for malignancy, *BRAF V600E*‐like signatures, and/or association with aggressive disease.^6^, ^7^, ^8^, ^9^, ^11^, ^12^, ^13^, ^25^, ^34^ In MPTX, all other mutations and fusions are categorized as weak oncogenic drivers based on literature supporting their presence in both benign and malignant thyroid nodules, their *RAS*‐like signatures, and/or the lack of literature supporting their high PPV for malignancy or aggressive behavior.^29^, ^35^, ^36^, ^37^, ^38^

### 2.4 Histopathology outcomes

Three pathologists each independently reviewed representative histopathology slides from surgically resected tissue of subjects. All three pathologists were blind to the histopathology diagnoses of the other pathologists and to MPTX results when their diagnosis was made. Unanimous consensus pathology diagnosis was defined by unanimous agreement between two nationally recognized pathologists with subspecialty expertise in thyroid pathology and the final local pathology at each institution. Histopathology results were categorized according to the World Health Organization (WHO) classification of tumors of the thyroid gland.^39^ Histopathology diagnoses of tumors with malignant behavior, as classified by the WHO, were considered malignant. Histopathology diagnoses of tumors with benign behavior, as classified by the WHO, were considered benign. All histopathology diagnoses were held in a secure, password protected database (OpenClinica) that was not accessible to those who performed molecular testing and finalized MPTX results.

### 2.5 Statistical analysis

Unblinding occurred by joining the final database (OpenClinica) of histopathology diagnoses to the final LIMS database of MPTX test results for analysis of test performance.

Diagnostic sensitivity, specificity, NPV, and PPV of the expanded mutation panel were determined using 2 × 2 contingency tables that compared negative (no mutations) and positive (any mutation) results to unanimous consensus histopathology diagnoses. For MPTX performance, diagnostic sensitivity was calculated at the threshold for negative MPTX results and diagnostic specificity was calculated at the threshold for positive MPTX results using 3 × 2 contingency tables comparing MPTX test results to unanimous consensus histopathology. The NPV of negative MPTX results, PPV of positive MPTX results, and rate of disease (ROD) in moderate MPTX results were determined using these 3 × 2 contingency tables. Moderate ROD is defined as the number of nodules with moderate MPTX test results that were malignant or NIFTP divided by the number of nodules with moderate MPTX test results.

Test performance was also examined after results of the study cohort were prevalence‐ adjusted to reflect the proportions of distinct histopathologic subtypes observed in a different study.^5^ Disease prevalence was assumed to be the same as that observed in our study. Proportions of malignant or NIFTP histopathologic subtypes were adjusted to include 14% (11/76) NIFTP, 13% (10/76) Hürthle cell carcinoma (HCC), 5% (4/76) follicular thyroid carcinoma (FTC), 64% (49/76) papillary thyroid carcinoma (PTC), and 3% (2/76) other malignancy types (Other M), as reported by Steward et al 2019. Proportions of benign nodule histopathologic subtypes were adjusted to include 55% (100/181) hyperplastic nodule (HN), 26% (47/181) follicular adenoma (FA), and 19% (34/181) Hürthle cell adenoma (HCA), as reported by Steward et al 2019. We then assumed that the observed probability (P) of being in a given test category (E) in a distinct histopathologic subtype (HS) would remain the same after the prevalence adjustment. A similar probability assumption is used in Bayes theorem to determine PPV over disease prevalence adjustments. P(E|HS) was derived from the mutation panel test data shown in Table S1. Table S2 demonstrates the application of P(E|HS) to the mutation panel data presented in Table S1 after the cohort was prevalence‐ adjusted to reflect the proportions of histopathologic subtypes reported by Steward et al 2019. For MPTX, P(E|HS) was derived from MPTX test data shown in Table S3 and applied to the prevalence‐ adjusted cohort in the same manner. After the prevalence adjustment, test performance characteristics were calculated using the same methods described above for calculating mutation panel and MPTX test performance.

Bayes theorem, P(H|E) = P(E|H)*P(H) / P(E|H)*P(H) + P(E|not H)*P(not H), was used to determine the expected NPV, PPV, and the ROD in moderate MPTX results over variable disease prevalence. P(H|E) is the probability of disease (H), including malignancy or NIFTP, in a given test category (E). P(E|H) is the probability of being in test category E in diseased subjects. P(H) is the prevalence of disease among all subjects. P(E|not H) is the probability of being in test category E in benign subjects (not H). P(not H) is the prevalence of benign disease among all subjects. Similarly, the rate at which each MPTX test category is expected to occur among all subjects tested over variable disease prevalence was also determined.^40^

## 3 RESULTS

### 3.1 Study cohort

**FIGURE 1:**
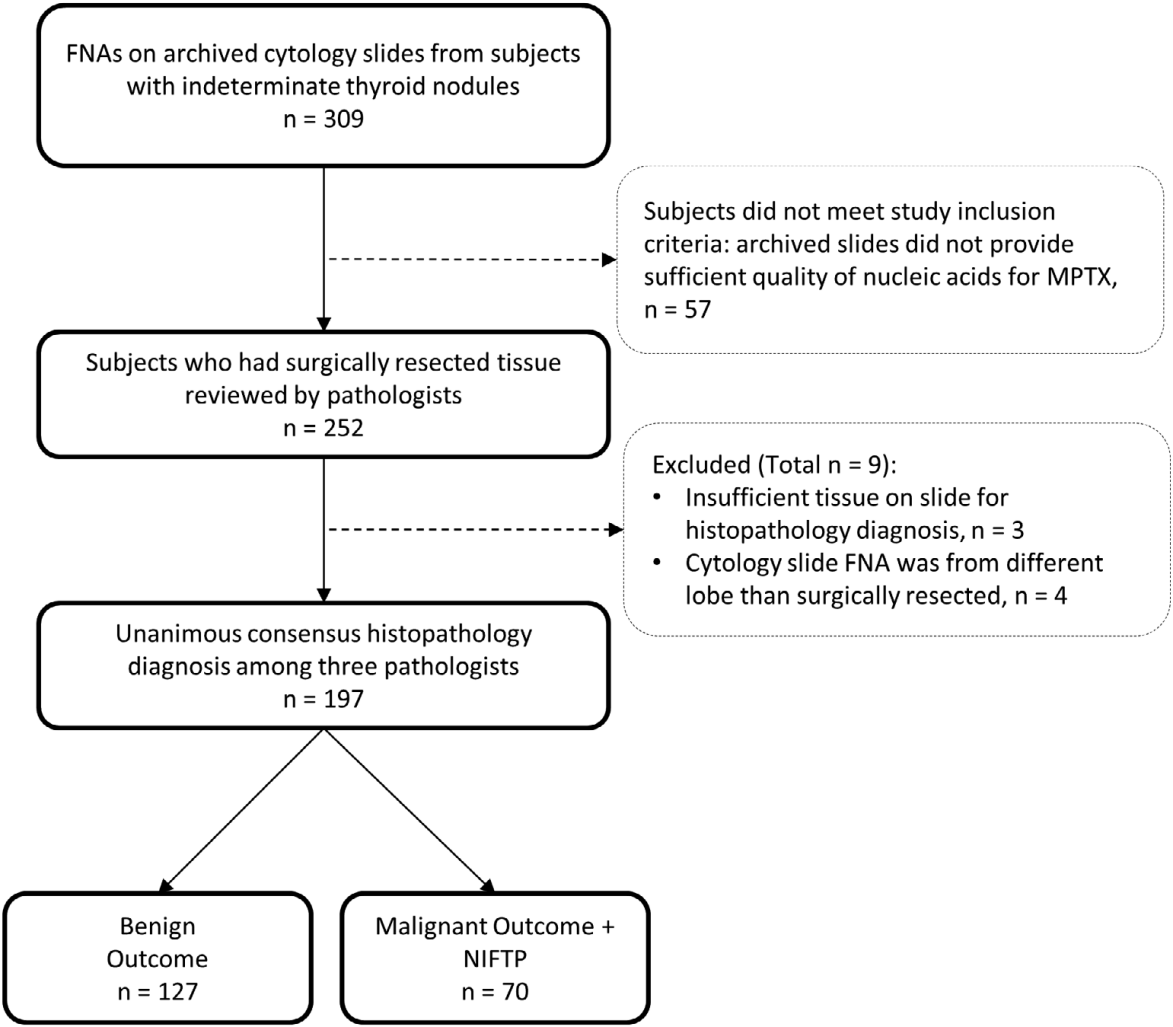
Breakdown of subjects in the study. Fine‐Needle Aspirate

Our multicenter study included 309 subjects with indeterminate thyroid nodules (Bethesda III, IV, or V) who had FNAs archived as cytology smear slides from nodules that later underwent surgical resection. Cytology slides provided by centers had been archived for a median of 3.1 years (range 0.7‐7.3 years) from FNA procedures that occurred between January 2013 and August 2019. A breakdown of subjects in the study is shown in Figure 1. Eighteen percent (57/309) of archived cytology slides did not meet study inclusion criteria, as they failed to provide sufficient nucleic acid quality for MPTX testing. This failure rate was consistent with that expected prior to the start of our study based on prestudy testing, where a 23% failure rate of cytology slides archived for a median of 5 years was observed. (SD Finkelstein, unpublished observation) Cytology slides were assessable by MPTX in a total of 252 subjects. Nine subjects were excluded due to discrepancies in surgical histopathology material provided (Figure 1). Unanimous consensus histopathology diagnosis was reached among three pathologists in 81% (197/243) of subjects. The majority (80%) of disagreement between pathologists was in the diagnosis of adenoma vs carcinoma. Subjects had a median age of 55 years (range 21‐87 years) and a median nodule size of 23 mm (range 3‐80 mm). The majority were female (73%) and had Bethesda III (47%) or Bethesda IV (44%) cytology. Only 10% had Bethesda V cytology. In total, 36% of subjects had disease, including malignancy or noninvasive follicular thyroid neoplasm with papillary‐like nuclear features (NIFTP).

### 3.2 Observed expanded mutation panel test performance

We first evaluated performance of the expanded mutation panel test by itself, without the addition of the microRNA risk classifier test (Table 1, ThyGeNEXT). Test performance observed in our study cohort is shown in Table 2 (n = 197; 36% disease prevalence). Notably, 90% (36/40) of all mutation panel false positive results were due to individual *RAS* mutations that were primarily found in benign adenomas (Table S1). Two additional false positive results were due to *TERT* promoter mutations that occurred in benign adenomas. Individual *RAS* mutations had a PPV of only 32%. *NRAS* mutations were the most frequently detected and had the highest PPV (37%) of all *RAS* mutation subtypes. Mutation panel false positive results occurred in a 33% (11/33) of Hürthle cell adenomas, with the majority (9/11) due to individual *RAS* mutations and one due to an individual *TERT* promoter mutation. By contrast, 75% (6/8) of Hürthle cell carcinomas had positive mutation panel results, with half (3/6) due to detection of *RAS* mutations that coexisted with *TERT* promoter mutations (Table S1).

**TABLE 2:** Mutation panel (ThyGeNEXT) test performance observed and mutation panel test performance after histopathologic subtype prevalence adjustment

Performance in Bethesda III, IV, and V nodules (n = 197, disease prevalence 36%)						
Mutation panel result	Benign	Malignant + NIFTP	Total	Parameter	Observed test performance	Prevalence adjusted test performance
	n	n	n		% (95% CI)	% (95% CI)
Negative	87	20	107	Sensitivity Specificity NPV PPV	71 (59‐82) 69 (60‐76) 81 (73‐88) 56 (45‐66)	74 (62‐84) 77 (69‐84) 84 (77‐91) 64 (53‐75)
Positive	40	50	90			
Total	127	70	197			

Abbreviations: NPV, negative predictive value; PPV, positive predictive value.

Given the high false positive rate of individual *RAS* mutations and resulting low PPV in our cohort, we verified the analytical accuracy of the next NGS based platform used for detecting *RAS* mutations. Thirty‐five *RAS* positive subjects, for which sufficient residual nucleic acid remained, were randomly selected and tested using a different analytical platform (ie, competitive allele‐specific PCR). There was 100% (95‐100%, 95%CI) qualitative agreement between the two platforms confirming the high analytical specificity of *RAS* testing using the NGS based platform.

### 3.3 Mutation panel test performance after histopathologic subtype prevalence adjustment

Compared to a recent prospective study of indeterminate thyroid nodules, our study cohort had a disproportionally large number of benign adenomas relative to hyperplastic nodules.^5^ Adenomas accounted for 81% of all benign nodules tested in our study (Table S1), while they accounted for less than half (45%) of benign nodules tested in the prospective study.^5^ Additional differences were also noted in the proportions of malignant or NIFTP subtypes in our study compared to that reported by Steward et al 2019. Due to these differences, we examined performance of the expanded mutation panel after proportions of these distinct histopathologic subtypes observed in our study were prevalence adjusted to match those reported by Steward el al 2019. The prevalence adjustment improved specificity and PPV of the mutation panel from 69% to 77% and from 56% to 64%, respectively (Table 2). It also marginally improved sensitivity and NPV. The PPV of individual *RAS* mutations increased from 32% to 46%, with *NRAS* having the highest PPV of all *RAS* mutation subtypes, which improved from 37% to 51% after the prevalence adjustment (Table S1 vs Table S2).

### 3.4 Observed MPTX test performance

We next evaluated the performance of MPTX, when the expanded mutation panel test was used in combination with the microRNA risk classifier test (Table 1, ThyGeNEXT and ThyraMIR) and results were categorized as negative, moderate, or positive. MPTX performance in various groups of cytologically indeterminate thyroid nodules observed in our study is shown in Table 3A‐D. Performance in Bethesda V nodules was not evaluated, as cases with this diagnoses were low (n = 19). Table S3A‐D shows the proportions of distinct histopathologic subtypes observed in our cohort for each Bethesda cytology group shown in Table 3A‐D.

**TABLE 3:** MPTX test performance observed and MPTX test performance after histopathologic subtype prevalence adjustment

A. Performance in Bethesda III and IV nodules (n = 178, disease prevalence 30%)					
MPTX result	Benign n	Malignant + NIFTP n	Parameter	Observed test performance, % (95% CI)	Prevalence adjusted test performance, % (95% CI)
Negative	77	4	Sensitivity Specificity NPV PPV Moderate ROD	93 (82‐98) Negative threshold 90 (84‐95) Positive threshold 95 (88‐99) 74 (60‐86) 30 (17‐44)	95 (86‐99) Negative threshold 90 (84‐95) Positive threshold 97 (91‐99) 75 (60‐86) 39 (32‐46)
Moderate	35	15			
Positive	12	35			

B. Performance in Bethesda III, IV, and V nodules (n = 197, disease prevalence 36%)					
MPTX result	Benign n	Malignant + NIFTP n	Parameter	Observed test performance, % (95% CI)	Prevalence adjusted test performance, % (95% CI)
Negative	78	4	Sensitivity Specificity NPV PPV Moderate ROD	94 (86‐98) Negative threshold 91 (84–95) Positive threshold 95 (88‐99) 81 (69‐90) 29 (17‐41)	96 (88‐99) Negative threshold 91 (84‐95) Positive threshold 97 (90‐99) 81 (70‐90) 33 (20‐47)
Moderate	37	15			
Positive	12	51			

C. Performance in Bethesda III nodules (n = 92, disease prevalence 36%)					
MPTX result	Benign n	Malignant + NIFTP n	Parameter	Observed test performance, % (95% CI)	Prevalence adjusted test performance, % (95% CI)
Negative	37	1	Sensitivity Specificity NPV PPV Moderate ROD	97 (84‐100) Negative threshold 93 (84‐98) Positive threshold 97 (86‐100) 85 (66‐96) 33 (16‐51)	97 (84‐100) Negative threshold 93 (84‐98) Positive threshold 98 (87‐100) 86 (75‐93) 38 (18‐58)
Moderate	18	9			
Positive	4	23			

D. Performance in Bethesda IV nodules (n = 86, disease prevalence 24%)					
MPTX result	Benign n	Malignant + NIFTP n	Parameter	Observed test performance, % (95% CI)	Prevalence adjusted test performance, % (95% CI)
Negative	40	3	Sensitivity Specificity NPV PPV Moderate ROD	86 (64‐97) Negative threshold 88 (77‐95) Positive threshold 93 (81‐99) 60 (36‐81) 26 (8‐44)	95 (76‐100) Negative threshold 88 (77‐95) Positive threshold 98 (89‐100) 64 (41‐83) 39 (29‐49)
Moderate	17	6			
Positive	8	12			

Abbreviations: NPV, negative predictive value; PPV, positive predictive value; ROD, rate of disease.

High sensitivity (95%) for malignancy was achieved at the threshold for negative MPTX results in nodules with Bethesda III and IV cytology (Table 3A; n = 178; 30% disease prevalence). High specificity (90%) was achieved at the threshold for positive MPTX results. As a result, MPTX had high NPV and PPV for malignancy. The ROD in moderate MPTX results was 30% (15/50) (Table 3A), and the majority of those results (76%) was attributed to detection of individual weak driver mutations that occurred in combination with negative or only moderate microRNA results, 95% (36/38) of which were individual *RAS* mutations (Table S4A). By contrast positive microRNA results were responsible for rulingin high risk of disease in 60% of MPTX positive nodules, 34% of which had individual *RAS* mutations (Table S4B). Positive microRNA results were also responsible for rulingin high risk of malignancy in one of four Hürthle cell carcinomas; the remaining three were ruledin by *TERT* promoter mutations that coexisted with *RAS* mutations. One of only four positive MPTX results in Hürthle cell adenomas was due to an individual *TERT* promoter mutation that did not coexist with other mutations.

### 3.5 MPTX test performance after histopathologic subtype prevalence adjustment

Table 3A‐D also shows performance of MPTX after the proportions of distinct histopathologic subtypes observed in our study were prevalence adjusted to match those reported by Steward el al 2019. Nearly all test performance parameters were similar or improved and there was no decrease in test performance after the prevalence adjustments were applied. Sensitivity and specificity of MPTX were similar among all Bethesda category subgroups examined after the prevalence adjustment. Prevalence adjustment had the highest impact on the performance of MPTX in Bethesda IV nodules, where before the adjustment, there was a relatively large proportion (88%) of benign nodules that were adenomas and a small proportion (43%) of malignant nodules that were papillary thyroid carcinomas (Table S3D). Sensitivity and NPV improved from 86% to 95% and from 93% to 98%, respectively (Table 3D).

**FIGURE 2:**
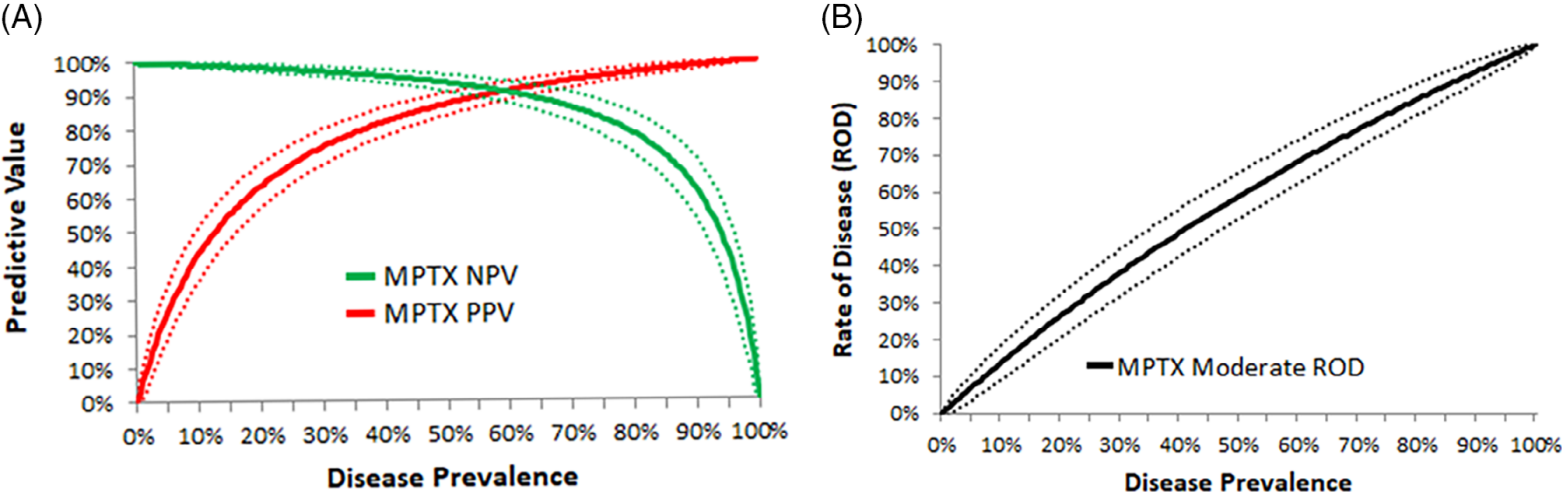
Expected performance of MPTX in Bethesda III and IV nodules over variable disease prevalence. A, The expected negative predictive value (NPV) and positive predictive value (PPV) of MPTX. B, The expected rate of disease (ROD) in moderate MPTX results. Dashed lines represent corresponding 95% confidence intervals

The expected NPV and PPV of MPTX and the expected ROD in moderate MPTX results based on prevalence adjusted performance in Bethesda III and IV nodules are shown over variable disease prevalence in Figure 2. At the highest disease prevalence expected in nodules with Bethesda IV cytology (40%), the NPV and PPV of MPTX was 96% and 82%, respectively (Figure 2A).^2^ Moderate MPTX results had a 49% ROD (Figure 2B). At the highest disease prevalence expected in nodules with Bethesda III cytology (30%), the NPV and PPV of MPTX was 97% and 75%, respectively.^2^ Moderate MPTX results had a 39% ROD.

**FIGURE 3:**
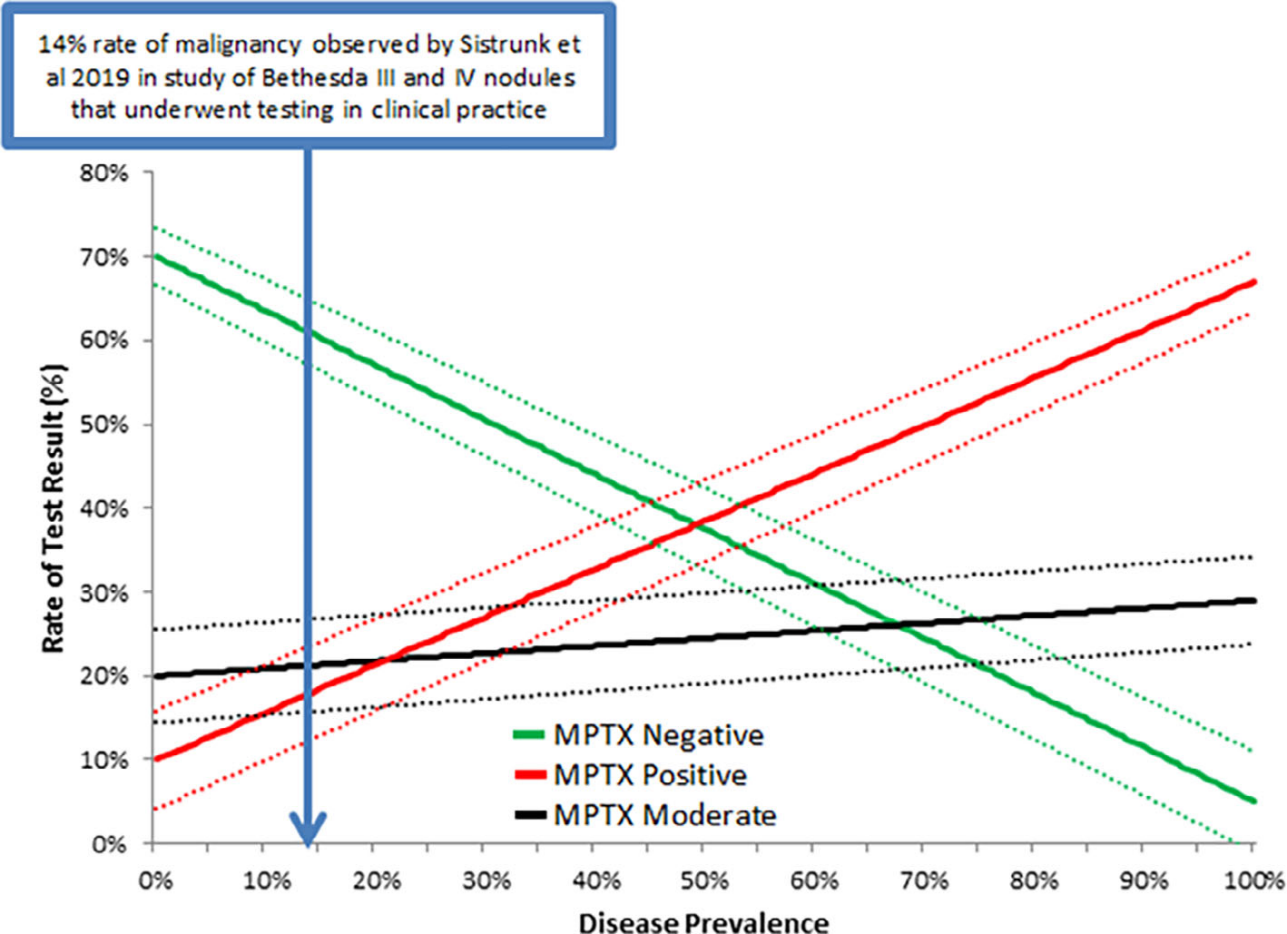
The rate at which negative, moderate, and positive MPTX results are expected to occur in clinically tested Bethesda III and IV nodules over variable disease prevalence. Dashed lines represent corresponding 95% confidence intervals

A prior observational study has shown that the rate of malignancy in Bethesda III and IV nodules that have undergone clinically prescribed molecular testing is only 14%.^19^ Since the ROD observed in our study was much higher for such nodules, we examined the expected rate at which each MPTX categorical result would occur at this lower disease prevalence (Figure 3). At a 14% rate of malignancy expected in clinical practice, 61% of nodules tested would have negative MPTX results, while 21% would have moderate MPTX results and 18% would have positive MPTX results. MPTX would accurately assist in rulingout and rulingin the need for surgery in 79% of cases. The remaining nodules would be assigned to the moderate MPTX category, wherein we have validated nodules have a moderate ROD primarily due to the presence of *RAS* mutations in adenomas where negative or moderate microRNA results cannot ruleout the need for surgery.

## 4 DISCUSSION

The objective of molecular testing of thyroid nodules with indeterminate cytology is to distinguish patients who are more likely to benefit from conservative management from those who are more likely to benefit from surgical intervention. We performed a blinded, multicenter clinical validation study of subjects who did not have past MPTX testing to evaluate if the multiplatform approach could assist in rulingin and rulingout the need for surgery. Since MPTX and other molecular tests with proven high NPV are currently used in clinical practice to help identify patients who should not undergo surgery, we feel that a retrospective study was a reasonable, ethical approach to obtaining the surgical histopathology reference standard required to assess MPTX test performance. We examined test performance in our study cohort and test performance adjusted to match the proportions of distinct histopathologic subtypes observed in a different prospective study.^5^ The gold standard comparator for test performance used was unanimous consensus histopathology diagnosis among three pathologists. Requiring this unanimous consensus ensured an accurate histopathology reference standard. The observed rates of unanimous consensus and inter‐observer variability among the three pathologists was consistent with that expected based on other reports.^31^, ^32^, ^41^

The expanded mutation panel test (ThyGeNEXT) by itself provides suboptimal NPV and PPV for malignancy when used without the microRNA risk classifier test. The suboptimal PPV was driven by individual *RAS* mutations that were detected in a large number of benign adenomas, which accounted for nearly all false positive test results. *NRAS* mutations were the most common. Others have also reported a low PPV for individual *RAS* mutations.^14^, ^15^, ^16^, ^29^, ^35^, ^36^, ^37^ Some have suggested that the variable PPV of *RAS* mutations reported across multiple studies may be due to differences in *RAS* performance in distinct histopathologic subtypes of benign and malignant disease.^16^ By applying prevalence adjustments, we have shown that all test performance parameters, including sensitivity and specificity, can be impacted by the distinct histopathologic subtypes used to evaluate test performance. Prevalence adjustments improved all test performance parameters of the mutation panel test and the PPV of individual *RAS* mutations. Given this, we suggest caution in interpreting the reported performance of tests that examine *RAS* and other mutations. Test performance can be misleading when cohorts are skewed toward distinct histopathologic subtypes in which tests have optimal specificity and/or sensitivity, such as in hyperplastic nodules and papillary thyroid carcinoma. Our results emphasize that decisions for surgery should not solely be made on the presence of *RAS* or *RAS*‐like mutations, given their high false positive rate in benign adenomas, which we confirmed in our study cohort using two different analytical platforms.

The MPTX multiplatform approach (ThyGeNEXT and ThyraMIR) provides optimal sensitivity, specificity, NPV, and PPV for malignancy. The high NPV of MPTX meets NCCN guideline requirements to consider nonsurgical treatment of thyroid nodules, where at least 95% NPV is necessary.^42^ The NPV and PPV of MPTX were similar to or exceeded that of other commercial tests that aim to ruleout and/or rulein high risk of disease in cytologically indeterminate thyroid nodules, where the need for surgery is uncertain due to moderate risk of malignancy.^5^, ^17^ Without ancillary molecular testing, the risk of malignancy is only 30% in Bethesda III nodules and 40% in Bethesda IV nodules at maximum. Negative MPTX results reduce this risk to only 3% to 4%, while positive MPTX results significantly elevate this risk to 75% to 82%. Based on our study, ancillary use of the three category MPTX approach is expected to accurately inform the need for surgery in four out of five indeterminate nodules tested. A similar finding was recently reported in indeterminate thyroid nodules that underwent MPTX testing in clinical practice.^30^ We have shown that these nodules include those with *RAS* mutations and those with Hürthle cells in which positive microRNA results and coexisting *TERT* mutations can help rulein the need for surgery.

Moderate MPTX results had a moderate ROD in our validation study. The majority of moderate results were due to nodules with weak driver mutations, primarily *RAS* mutations, in which microRNA results were negative or only moderate, where we and others have also shown that cancer cannot be ruledout.^19^, ^29^ Given the moderate rate of disease validated herein, patients may benefit from close surveillance, depending on other clinical factors, such as personal and family histories, nodule sonographic features, thyroid function tests, and patient preference. Although close surveillance is an option, lobectomy can be justified given that the majority of these cases will be neoplastic adenomas. Lobectomy may be more appropriate when Hürthle cells are observed in FNAs, as moderate MPTX results cannot exclude the possibility of Hürthle cell carcinoma.

In addition to validating the performance of the MPTX multiplatform approach, our study supports the utility of mutation panels that include key therapeutic and prognostic markers. An *NTRK3* fusion, which is a known therapeutic target, was detected in one nodule with malignancy (Table S1). Multiple coexisting mutations were also detected. All coexisting mutations occurred in malignant nodules and were paired with *TERT* promoter mutations. Such coexisting mutations have the potential to promote aggressive tumor behavior and have been associated with poor patient survival.^9^, ^10^, ^11^, ^43^ Consistently, coexisting mutations were found in aggressive cancer types, including Hürthle cell carcinoma, poorly differentiated thyroid carcinoma, and widely invasive follicular thyroid carcinoma (Table S1). Remarkably, two subjects had benign adenomas with individual *TERT* promoter mutations that did not coexist with other mutations. Although infrequent, others have detected *TERT* mutations in follicular adenomas.^44^, ^45^, ^46^ Some have suggested that these mutations may be an early genetic event in follicular tumors that have yet to show morphological signs of malignancy.^46^ However, results of these studies and ours highlight the need to further validate the predictive value of individual *TERT* mutations.

Our study is the first to validate the clinical performance of MPTX in a well‐controlled study where unanimous consensus histopathology among three independent pathologists was used as the gold standard for test performance. A limitation of our study was that MPTX performance was evaluated in FNAs preserved as cytology slides that were archived for up to 7 years, during which time DNA and RNA can degrade. As expected, some cytology slides were not assessable in our study due to this. In our experience, only 5.0% (11/218) of cytology slides fail MPTX testing in clinical practice.(N Massoll, unpublished observation) Similarly, a recent large study of over 4000 patients reported that only 4.5% of cytology smears and direct aspirates, for which there is 90% to 98% concordance between MPTX results, fail molecular testing in clinical practice.^21^ In addition, our study had a large number of benign adenomas. To address this, MPTX test performance observed in our study was prevalence adjusted to match the proportions of distinct histopathologic subtypes observed in a recent prospective study in which adenomas were less frequent.^5^ After the prevalence adjustment, MPTX test performance was similar or improved. We also did not encounter any cases of medullary thyroid carcinoma (MTC) in our study, and consequently we were unable to further validate the reported utility of using the mutation panel in combination with microRNA analysis to identify MTC.^47^, ^48^

Our results emphasize that decisions for surgery should not solely be made on the presence of *RAS* mutations, given their high false positive rate in benign adenomas. Additional studies that validate the PPV of individual weak driver, *RAS*‐like mutations in cohorts that better reflect all distinct histopathologic subtypes of benign and malignant disease are needed before such mutations are solely used in surgical decision making. The common occurrence of *RAS* mutations in benign adenomas lowers the PPV of molecular tests that report binary results. Nodules with *RAS* mutations are a frequent clinical scenario and a circumstance in which additional molecular, imaging, and clinical features are needed to help guide decisions. One of the unique utilities of the microRNA classifier test is its ability to rulein high risk disease in nodules with *RAS* mutations. Although MPTX can rulein high risk when *RAS* is detected, it is less effective at rulingout disease in these cases, and as a result a small portion of nodules that have moderate risk of malignancy will be reported as such in clinical practice.

## CONFLICT OF INTEREST

J. Woody Sistrunk, Nicole Massoll, Ryan Campbell, Ann E. Walts, and Shikha Bose received institutional research funding from Interpace Diagnostics. Mark A. Lupo has received research funding from Interpace Diagnostics. J. Woody Sistrunk and Nicole Massoll are consultants for Interpace Diagnostics. Histopathology review by Thomas J. Giordano and Peter M. Sadow was funded by Interpace Diagnostics. Sara A. Jackson, Christina M. Narick, Nicole Toney, Sydney D. Finkelstein, Alidad Mireskandari, Gyanendra Kumar are employees of Interpace Diagnostics.

## AUTHOR CONTRIBUTIONS

Mark A. Lupo, Ann E. Walts, J. Woody Sistrunk, Nicole Massoll, Ryan Campbell, Sara A. Jackson, Nicole Toney, Christina M. Narick, Gyanendra Kumar, Alidad Mireskandari, Sydney D. Finkelstein, and Shikha Bose each participated in study design, generating data for the study, review of data analysis, and preparation of the manuscript into its final form. Thomas J. Giordano and Peter M. Sadow provided histopathology diagnoses, reviewed data analysis, and participated in preparation of the manuscript into its final form. All authors approved the manuscript in its final form prior to submission.

## Supporting information


**Table S1** Performance of the mutation panel test observed with the proportions of each distinct histopathologic (histo.) subtype observed shown.

**Table S2** Performance of the mutation panel test after histopathologic (histo.) subtype prevalence adjustment, with numbers rounded to one decimal shown.

**Table S3** Performance of MPTX observed with the proportions of each distinct histopathologic (histo.) subtype observed shown.

**Table S4** Positive and moderate MPTX results in Bethesda III or IV nodules observed broken down by mutation panel result and microRNA risk classifier result.
