# Supplementary material for: Multiplatform molecular test performance in indeterminate thyroid nodules
Source: Diagn Cytopathol. 2020 Aug 7;48(12):1254–64. doi: 10.1002/dc.24564 (PMC7754490; doi:10.1002/dc.24564)
Supplement: Supplementary file 1 — Table S1 Performance of the mutation panel test observed with the proportions of each distinct histopathologic (histo.) subtype observed shown. [file DC-48-1254-s001.docx]

| Supplementary Table 1. Performance of the mutation panel test observed with the proportions of each distinct histopathologic (histo.) subtype observed shown. | | | | | | | | | | | | | | | |
| --- | --- | --- | --- | --- | --- | --- | --- | --- | --- | --- | --- | --- | --- | --- | --- |
| **Performance in Bethesda III, IV, and V nodules (N = 197, disease prevalence 36%)** | | | | | | | | | | | | | | | |
|  | | | **% Benign histo. subtype** | | |  | **% Malignant or NIFTP histo. subtype disease** | | | | |  |  |  |  |
|  | | | **19%** | **55%** | **26%** |  | **7%** | **11%** | **10%** | **70%** | **1%** |  | **Total** | **ROD** | **Test Performance** |
|  | | | **HN** | **FA** | **HCA** |  | **NIFTP** | **HCC** | **FTC** | **PTC** | **PDTC** |  |  |  |  |
| **Mutation Panel Result** | | | **N** | **N** | **N** |  | **N** | **N** | **N** | **N** | **N** |  | **N** | **%** | **% (95% CI)** |
| **Negative** | | | **21** | **44** | **22** |  | **0** | **2** | **2** | **16** | **0** |  | **107** | **19** | Se, 71 (59-82) |
| **Positive** | | | **3** | **26** | **11** |  | **5** | **6** | **5** | **33** | **1** |  | **90** | **56** | Sp, 69 (60-76) |
|  | *RAS* | |  |  |  |  |  |  |  |  |  |  |  | 32 | NPV, 81 (73-88) |
|  |  | *NRAS* | 1 | 15 | 3 |  | 3 | 1 | 2 | 5 | 0 |  | 30 | 37 | PPV, 56 (45-66) |
|  |  | *KRAS* | 1 | 5 | 3 |  | 1 | 1 | 0 | 1 | 0 |  | 12 | 25 |  |
|  |  | *HRAS* | 0 | 5 | 3 |  | 1 | 0 | 1 | 1 | 0 |  | 11 | 27 |  |
|  | *BRAF K601E* | | 1 | 0 | 0 |  | 0 | 0 | 0 | 0 | 0 |  | 1 | 0 |  |
|  | *PPARg* fusion | | 0 | 0 | 1 |  | 0 | 1 | 0 | 0 | 0 |  | 2 | 50 |  |
|  | *TERT* | | 0 | 1 | 1 |  | 0 | 0 | 1**^‡^** | 0 | 0 |  | 3 | 33 |  |
|  | *TERT, RAS* | | 0 | 0 | 0 |  | 0 | 2 | 1 | 0 | 1 |  | 4 | 100 |  |
|  | *PIK3CA, TERT, RAS* | | 0 | 0 | 0 |  | 0 | 1 | 0 | 0 | 0 |  | 1 | 100 |  |
|  | *NTRK* fusion | | 0 | 0 | 0 |  | 0 | 0 | 0 | 1 | 0 |  | 1 | 100 |  |
|  | *RET* fusion | | 0 | 0 | 0 |  | 0 | 0 | 0 | 2 | 0 |  | 2 | 100 |  |
|  | *BRAF V600E* | | 0 | 0 | 0 |  | 0 | 0 | 0 | 22 | 0 |  | 22 | 100 |  |
|  | *BRAF* fusion | | 0 | 0 | 0 |  | 0 | 0 | 0 | 1 | 0 |  | 1 | 100 |  |
| **Total** | | | **24** | **70** | **33** |  | **5** | **8** | **7** | **49** | **1** |  | **197** | **36** |  |
| ^‡^ Widely invasive FTC | | | | | | | | | | | | | | | |
| Hyperplastic Nodule (HN), Follicular Adenoma (FA), and Hürthle Cell Adenoma (HCA), Noninvasive Follicular Thyroid Neoplasm with Papillary-like Nuclear Features (NIFTP), Hürthle Cell Carcinoma (HCC), Follicular Thyroid Carcinoma (FTC), Papillary Thyroid Carcinoma (PTC), Poorly Differentiated Thyroid Carcinoma (PDTC); Sensitivity (Se), Specificity (Sp), negative predictive value (NPV), positive predictive value (PPV); histopathologic (histo.); rate of disease (ROD) | | | | | | | | | | | | | | | |
