# Supplementary material for: Multiplatform molecular test performance in indeterminate thyroid nodules
Source: Diagn Cytopathol. 2020 Aug 7;48(12):1254–64. doi: 10.1002/dc.24564 (PMC7754490; doi:10.1002/dc.24564)
Supplement: Supplementary file 2 — Table S2 Performance of the mutation panel test after histopathologic (histo.) subtype prevalence adjustment, with numbers rounded to one decimal shown. [file DC-48-1254-s002.docx]

| Supplementary Table 2. Performance of the mutation panel test after histopathologic (histo.) subtype prevalence adjustment, with numbers rounded to one decimal shown. | | | | | | | | | | | | | | | |
| --- | --- | --- | --- | --- | --- | --- | --- | --- | --- | --- | --- | --- | --- | --- | --- |
| **Performance of mutation panel in Bethesda III, IV, and V nodules (N = 197, disease prevalence 36%)** | | | | | | | | | | | | | | |  |
|  | | | **% Benign histo. subtype** | | |  | **% Malignant or NIFTP histo. subtype disease** | | | | |  |  | **Prevalence Adjusted** | **Prevalence Adjusted** |
|  | | | **55.2%** | **26.0%** | **18.8%** |  | **14.5%** | **13.2%** | **5.3%** | **64.5%** | **2.6%** |  | **Total** | **ROD** | **Test Performance** |
|  | | | **HN** | **FA** | **HCA** |  | **NIFTP** | **HCC** | **FTC** | **PTC** | **Other M^†^** |  |  |  |  |
| **Mutation Panel Result** | | | **N** | **N** | **N** |  | **N** | **N** | **N** | **N** | **N** |  | **N** | **%** | **% (95% CI)** |
| **Negative** | | | **61.4** | **20.7** | **15.9** |  | **0.0** | **2.3** | **1.1** | **14.7** | **0.0** |  | **116.1** | **16** | Se, 74 (62-84) |
| **Positive** | | | **8.8** | **12.2** | **8.0** |  | **10.1** | **6.9** | **2.6** | **30.4** | **1.8** |  | **80.9** | **64** | Sp, 77 (69-84) |
|  | *RAS* | |  |  |  |  |  |  |  |  |  |  |  | 46 | NPV, 84 (77-91) |
|  |  | *NRAS* | 2.9 | 7.1 | 2.2 |  | 6.1 | 1.2 | 1.1 | 4.6 | 0.0 |  | 25.0 | 51 | PPV, 64 (53-75) |
|  |  | *KRAS* | 2.9 | 2.4 | 2.2 |  | 2.0 | 1.2 | 0.0 | 0.9 | 0.0 |  | 11.5 | 35 |  |
|  |  | *HRAS* | 0.0 | 2.4 | 2.2 |  | 2.0 | 0.0 | 0.5 | 0.9 | 0.0 |  | 8.0 | 43 |  |
|  | *BRAF K601E* | | 2.9 | 0.0 | 0.0 |  | 0.0 | 0.0 | 0.0 | 0.0 | 0.0 |  | 2.9 | 0 |  |
|  | *PPARg* fusion | | 0.0 | 0.0 | 0.7 |  | 0.0 | 1.2 | 0.0 | 0.0 | 0.0 |  | 1.9 | 61 |  |
|  | *TERT* | | 0.0 | 0.5 | 0.7 |  | 0.0 | 0.0 | 0.5 | 0.0 | 0.0 |  | 1.7 | 31 |  |
|  | *TERT, RAS* | | 0.0 | 0.0 | 0.0 |  | 0.0 | 2.3 | 0.5 | 0.0 | 1.8 |  | 4.6 | 100 |  |
|  | *PIK3CA, TERT, RAS* | | 0.0 | 0.0 | 0.0 |  | 0.0 | 1.2 | 0.0 | 0.0 | 0.0 |  | 1.2 | 100 |  |
|  | *NTRK* fusion | | 0.0 | 0.0 | 0.0 |  | 0.0 | 0.0 | 0.0 | 0.9 | 0.0 |  | 0.9 | 100 |  |
|  | *RET* fusion | | 0.0 | 0.0 | 0.0 |  | 0.0 | 0.0 | 0.0 | 1.8 | 0.0 |  | 1.8 | 100 |  |
|  | *BRAF V600E* | | 0.0 | 0.0 | 0.0 |  | 0.0 | 0.0 | 0.0 | 20.3 | 0.0 |  | 20.3 | 100 |  |
|  | *BRAF* fusion | | 0.0 | 0.0 | 0.0 |  | 0.0 | 0.0 | 0.0 | 0.9 | 0.0 |  | 0.9 | 100 |  |
| **Total** | | | **70.2** | **33.0** | **23.9** |  | **10.1** | **9.2** | **3.7** | **45.1** | **1.8** |  | **197.0** | **36** |  |
| **^†^**Other M (Malignancy) was poorly differentiated thyroid carcinoma in our study, while it was Medullary thyroid carcinoma in Steward et al 2019 | | | | | | | | | | | | | | | |
| Hyperplastic Nodule (HN), Follicular Adenoma (FA), and Hürthle Cell Adenoma (HCA), Noninvasive Follicular Thyroid Neoplasm with Papillary-like Nuclear Features (NIFTP), Hürthle Cell Carcinoma (HCC), Follicular Thyroid Carcinoma (FTC), Papillary Thyroid Carcinoma (PTC), other Malignancy (Other M); Sensitivity (Se), Specificity (Sp), negative predictive value (NPV), positive predictive value (PPV); histopathologic (histo.); rate of disease (ROD) | | | | | | | | | | | | | | | |
