# Supplementary material for: Multiplatform molecular test performance in indeterminate thyroid nodules
Source: Diagn Cytopathol. 2020 Aug 7;48(12):1254–64. doi: 10.1002/dc.24564 (PMC7754490; doi:10.1002/dc.24564)
Supplement: Supplementary file 3 — Table S3 Performance of MPTX observed with the proportions of each distinct histopathologic (histo.) subtype observed shown. [file DC-48-1254-s003.docx]

| Supplementary Table 3. Performance of MPTX observed with the proportions of each distinct histopathologic (histo.) subtype observed shown. | | | | | | | | | | |
| --- | --- | --- | --- | --- | --- | --- | --- | --- | --- | --- |
| **A. Performance in Bethesda III and IV nodules (N = 178, disease prevalence 30%)** | | | | | | | | | | |
|  | **% Benign histo. subtype** | | | **% Malignant or NIFTP histo. subtype disease** | | | | | **Total** | **Test Performance** |
|  | **18%** | **56%** | **27%** | **7%** | **15%** | **13%** | **63%** | **2%** |  | **% (95% CI)** |
| **MPTX Result** | **HN** | **FA** | **HCA** | **NIFTP** | **HCC** | **FTC** | **PTC** | **PDTC** |  | Se, 93 (82-98) Neg Thr  Sp, 90 (84-95) Pos Thr  NPV, 95 (88-99)  PPV, 74 (60-86)  Moderate ROD, 30 (17-44) |
|  | **N** | **N** | **N** | **N** | **N** | **N** | **N** | **N** | **N** |  |
| **Negative** | 18 | 42 | 17 | 0 | 2 | 2 | 0 | 0 | 81 |  |
| **Moderate** | 2 | 21 | 12 | 1 | 2 | 1 | 11 | 0 | 50 |  |
| **Positive** | 2 | 6 | 4 | 3 | 4 | 4 | 23 | 1 | 47 |  |
| **B. Performance in Bethesda III, IV, and V nodules (N = 197, disease prevalence 36%)** | | | | | | | | | | |
|  | **% Benign histo. subtype** | | | **% Malignant or NIFTP histo. subtype disease** | | | | | **Total** | **Test Performance** |
|  | **19%** | **55%** | **26%** | **7%** | **11%** | **10%** | **70%** | **1%** |  | **% (95% CI)** |
| **MPTX Result** | **HN** | **FA** | **HCA** | **NIFTP** | **HCC** | **FTC** | **PTC** | **PDTC** |  | Se, 94 (86-98) Neg Thr  Sp, 91 (84-95) Pos Thr  NPV, 95 (88-99)  PPV, 81 (69-90)  Moderate ROD, 29 (17-41) |
|  | **N** | **N** | **N** | **N** | **N** | **N** | **N** | **N** | **N** |  |
| **Negative** | 18 | 43 | 17 | 0 | 2 | 2 | 0 | 0 | 82 |  |
| **Moderate** | 4 | 21 | 12 | 1 | 2 | 1 | 11 | 0 | 52 |  |
| **Positive** | 2 | 6 | 4 | 4 | 4 | 4 | 38 | 1 | 63 |  |
| **C. Performance in Bethesda III nodules (N = 92, disease prevalence 36%)** | | | | | | | | | | |
|  | **% Benign histo. subtype** | | | **% Malignant or NIFTP histo. subtype disease** | | | | | **Total** | **Test Performance** |
|  | **24%** | **51%** | **25%** | **9%** | **6%** | **9%** | **76%** | **0%** |  | **% (95% CI)** |
| **MPTX Result** | **HN** | **FA** | **HCA** | **NIFTP** | **HCC** | **FTC** | **PTC** | **PDTC** |  | Se, 97 (84-100) Neg Thr  Sp, 93 (84-98) Pos Thr  NPV, 97 (86-100)  PPV, 85 (66-96)  Moderate ROD, 33 (16-51) |
|  | **N** | **N** | **N** | **N** | **N** | **N** | **N** | **N** | **N** |  |
| **Negative** | 11 | 16 | 10 | 0 | 0 | 1 | 0 | 0 | 38 |  |
| **Moderate** | 2 | 11 | 5 | 1 | 0 | 0 | 8 | 0 | 27 |  |
| **Positive** | 1 | 3 | 0 | 2 | 2 | 2 | 17 | 0 | 27 |  |
| **D. Performance in Bethesda IV nodules (N = 86, disease prevalence 24%)** | | | | | | | | | | |
|  | **% Benign histo. subtype** | | | **% Malignant or NIFTP histo. subtype disease** | | | | | **Total** | **Test Performance** |
|  | **12%** | **60%** | **28%** | **5%** | **29%** | **19%** | **43%** | **5%** |  | **% (95% CI)** |
| **MPTX Result** | **HN** | **FA** | **HCA** | **NIFTP** | **HCC** | **FTC** | **PTC** | **PDTC** |  | Se, 86 (64-97) Neg Thr  Sp, 88 (77-95) Pos Thr  NPV, 93 (81-99)  PPV, 60 (36-81)  Moderate ROD, 26 (8-44) |
|  | **N** | **N** | **N** | **N** | **N** | **N** | **N** | **N** | **N** |  |
| **Negative** | 7 | 26 | 7 | 0 | 2 | 1 | 0 | 0 | 43 |  |
| **Moderate** | 0 | 10 | 7 | 0 | 2 | 1 | 3 | 0 | 23 |  |
| **Positive** | 1 | 3 | 4 | 1 | 2 | 2 | 6 | 1 | 20 |  |
| Hyperplastic Nodule (HN), Follicular Adenoma (FA), and Hürthle Cell Adenoma (HCA), Noninvasive Follicular Thyroid Neoplasm with Papillary-like Nuclear Features (NIFTP), Hürthle Cell Carcinoma (HCC), Follicular Thyroid Carcinoma (FTC), Papillary Thyroid Carcinoma (PTC), Poorly Differentiated Thyroid Carcinoma (PDTC), Sensitivity (Se), Specificity (Sp), negative predictive value (NPV), positive predictive value (PPV); negative threshold of MPTX (Neg Thr); positive threshold of MPTX (Pos Thr); histopathologic (histo.); rate of disease (ROD) | | | | | | | | | | |
