# Supplementary material for: Multiplatform molecular test performance in indeterminate thyroid nodules
Source: Diagn Cytopathol. 2020 Aug 7;48(12):1254–64. doi: 10.1002/dc.24564 (PMC7754490; doi:10.1002/dc.24564)
Supplement: Supplementary file 4 — Table S4 Positive and moderate MPTX results in Bethesda III or IV nodules observed broken down by mutation panel result and microRNA risk classifier result. [file DC-48-1254-s004.docx]

| Supplementary Table 4. Positive and moderate MPTX results in Bethesda III or IV nodules observed broken down by mutation panel result and microRNA risk classifier result. | | | |
| --- | --- | --- | --- |
| 1. **Moderate MPTX Result (N=50)** | **Mutation Panel Result** | **MicroRNA Classifier Result** | **N (% Total)** |
|  | Weak driver (*RAS*) | Negative or moderate | 36 (72%) |
|  | Weak driver (*PPARg* fusion) | Negative or moderate | 2 (4%) |
|  | No mutations | Moderate | 12 (24%) |
|  |  |  |  |
| 1. **Positive MPTX Result (N=47)** | **Mutation Panel Result** | **MicroRNA Classifier Result** | **N (% Total)** |
|  | No mutations | Positive | 11 (23%) |
|  | Weak driver (*RAS*) | Positive | 16 (34%) |
|  | Weak driver (*NTRK* fusion) | Positive | 1 (2%) |
|  | Strong Driver | Not performed | 19 (40%) |
